# The alteration from agricultural to nomadic regimes resulted in human livelihood transformation in North-Central China during the 12th century: The archaeobotanical evidence

**DOI:** 10.3389/fpls.2022.978147

**Published:** 2022-09-16

**Authors:** Ruo Li, Bing Li, Wei Chen, Peilun Liu, Mingxia Xie, Yunqing Zhang, Sai Wang, Yuecong Li, Guanghui Dong

**Affiliations:** ^1^Key Laboratory of Western China’s Environmental Systems (Ministry of Education), College of Earth and Environmental Sciences, Lanzhou University, Lanzhou, China; ^2^School of Geographical Sciences, Hebei Normal University, Shijiazhuang, China; ^3^Hebei Provincial Institute of Cultural Relics and Archaeology, Shijiazhuang, Hebei, China; ^4^Hebei Key Laboratory of Environmental Change and Ecological Construction, Shijiazhuang, China

**Keywords:** archaeobotanical analysis, North-central China, subsistence strategy, the 12th century, Northern Song Dynasty, Jin Dynasty, geopolitical change

## Abstract

Human livelihoods provided a crucial economic foundation for social development in ancient times and were influenced by various factors including environmental change, agricultural origin and intensification, as well as long-distance exchange and culinary tradition. The effect of geopolitical change on human subsistence, especially the shifts between agricultural and nomadic regimes, has not been well understood due to the absence of detailed historical records and archaeological evidence. During the 12th century, the control of the Zhengding area in Hebei Province of north-central China changed from the Northern Song (960–1127 CE) to the *Jurchen* Jin Dynasty (Jin Dynasty; 1115–1234 CE). Recent excavation of the Zhengding Kaiyuan Temple South (ZKS) site in the area provides a rare opportunity to study human livelihood transformation in relation to geopolitical change. In total, 21,588 charred crop caryopses including foxtail millet, wheat, broomcorn millet, hulled barley, and rice, and other carbonized remains including 55.15 g of boiled foxtail millet and 353.5 g of foxtail millet caryopses were identified, and nine AMS ^14^C dates of crop remains were obtained from the Northern Song and Jin layers at the ZKS site. This revealed that the dominant plant subsistence transformed from wheat to foxtail millet during the change from the Northern Song to the Jin Dynasties in Zhengding area. By comparing with historical documents and paleoclimate records, we propose that this abnormal shift of primary staple food from the relatively high-yield wheat to low-yield foxtail millet was induced by the traditional dietary preference for foxtail millet in the nomadic Jin society. The Jin government levied foxtail millet as taxation and promoted massive immigration from northeastern China to north-central China to consolidate their rule, which resulted in the adoption of foxtail millet as the most important crop in Zhengding area. The advantage for the cultivation of this frost-sensitive crop in north-central China over northeast China was probably enhanced by notable cold events during the 12th century, while the primary influencing factor for the transformation of human livelihoods in north-central China during that period was geopolitics rather than climate change.

## Introduction

The origin and spread of agriculture provided a stable economic foundation for the development of human societies and the establishment of ancient civilizations (Diamond and Bellwood, 2003; Iriarte et al., 2004; Chen et al., 2015a; Yang et al., 2022). China has long been accepted as one of the three independent world centers for the origin of crop-based agriculture (Zhao and Piperno, 2000; Fuller et al., 2009; Lu et al., 2009; Yang et al., 2012; Zuo et al., 2017). With methodological advances in archaeobotany such as flotation and radiocarbon dating, the development of agriculture and its influencing factors in prehistoric China have been intensively studied (He et al., 2017; Long et al., 2018; Li R. et al., 2020). Originating in the Yellow River basin in the early Holocene, millet agriculture (based on both foxtail and broomcorn millets) was established around 4000 BCE in northern China (Barton et al., 2009; Zhao, 2014). After the introduction of west Asian wheat in the third millennium, northern Chinese agriculture gradually transitioned from millet-based to wheat-based (Dodson et al., 2013; Dong G. H. et al., 2017; Zhang et al., 2017; Long et al., 2018; Liu et al., 2019). Scholars have demonstrated that the adoption of wheat in agriculture showed spatial-temporal variation before the Han Dynasty (202 BCE–220 CE), which was primarily promoted by climate change and technological innovation (Ma et al., 2016; Zhou et al., 2016; Dong Y. et al., 2017; Zhou et al., 2017). However, the development of agriculture in historical China, especially the way it transitioned, is still poorly understood.

The transformation of agriculture in historical China is more complex and has been widely debated (Han, 1999; Wang, 2016; Tian and Zhou, 2020; Zhao, 2020). According to historical documents, the agricultural pattern in China has changed from “rice in the south and foxtail millet in the north” to “rice in the south and wheat in the north,” with controversies remaining as to when and how it took place when based only on written records (Wang, 2000, 2016; Han, 2013). The archaeobotanical analysis is an important method for understanding the historical evolution of agriculture and has been carried out in different parts of the world during the historical periods, bridging the gaps in studies of ancient agriculture (Kennett and Marwan, 2015; Zhao, 2020). Previous interdisciplinary studies have proposed that geopolitics replaced climate change as the key driver in historical changes in agriculture (Shi et al., 2022; Wilson et al., 2022), such as those in the Central Andes (Kennett and Marwan, 2015; Wilson et al., 2022), in Europe (Messer, 1997), and northwestern China (Shi et al., 2022). Nevertheless, this type of interdisciplinary investigation has not focused on north-central China, due to the absence of detailed archaeobotanical evidence and accurate dating.

North-central China is located in the monsoon region and is sensitive to climate change, with good conditions for agricultural development due to the fertile soil and simultaneous rain and heat. As a dominant agricultural region close to nomadic areas, north-central China has been controlled by agrarian and nomadic regimes and characterized by complex geopolitical features throughout historical times. Thus, it is an ideal region to explore the transformation of agriculture and its influencing factors in the historical period. In the 10th–13th centuries, several powerful regimes coexisted in Northern China, including the nomadic regimes of Liao (907–1125 CE), Jin (1115–1234 CE), and Xixia (1038–1227 CE); and the agrarian regime of the Northern Song (960–1127 CE). This resulted in frequent conflicts, and eventually, Jin became the only nomadic regime occupying this agricultural region. However, it remains unclear whether the frequent changes in geopolitical patterns, especially the conversion of the agricultural Northern Song and the pastoralist Jin regimes, have influenced the agricultural economy in north-central China in the 10th–13th centuries.

The ZKS site is located in Zhengding County, Hebei Province, which is an important agricultural region close to the Sixteen Prefectures of Yanyun (*燕云十六州*, the natural barrier between agrarian regimes and nomadic regimes in northern China). It experienced regime change from the Northern Song to Jin in the 12th century and there are clear cultural layers of the two regimes at the site. The recent excavation, therefore, provides an excellent opportunity to explore how geopolitical patterns and climate change have influenced the development of agriculture in north-central China during the 10th–13th centuries. This paper is concerned with the identification of carbonized crop remains from the ZKS site and an accurate sequence based on the radiocarbon dating of short-lived crop caryopses, which was compared to the ceramic dates from the site and written records about Zhengding. In addition, we have used other archaeological data, and historical analysis including agricultural history and phenological events (Ge et al., 2003), as well as a paleoclimate record from lacustrine deposits of the Gonghai Lake in Northern China (Chen et al., 2015b). Together, this data allows us to examine the spatio-temporal variation of agriculture and to evaluate the natural and geopolitical factors influencing the changes to human livelihoods.

## Study area and site background

**FIGURE 1:**
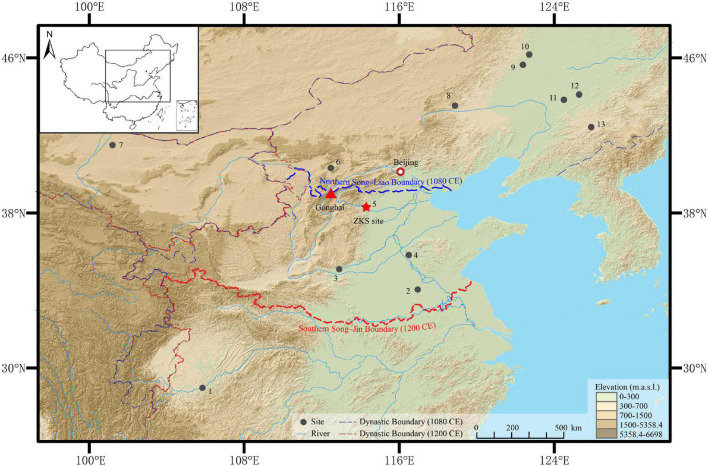
The location of the study area and the sites mentioned in the text (Digital Elevation Model, http://www.gscloud.cn; approximate shapes of Chinese dynasties https://sites.fas.harvard.edu/~chgis/data): 1. Handong Cheng (Ma et al., 2020); 2. Oupanyao (Bai et al., 2017); 3. Xijincheng (Chen et al., 2010); 4. Liang Zhuang (Wu et al., 2010); 5. ZKS (this study); 6. Zhuangwocun (Zhao, 2020); 7. Heicheng (Shi et al., 2022); 8. Bayantala (Sun and Zhao, 2014); 9. Sun Changqing (Yang et al., 2010a); 10. Yongping (Yang, 2014a); 11. Changshan (Chen, 2019); 12. Lichunjiang (Yang et al., 2010b); 13. Luotongshancheng (Yang, 2014b).

Zhengding County, Hebei Province is in the middle and upper proluvial-alluvial plain in front of the Taihang mountain, with the Hutuo river crossing to the south (Figure 1). In Zhengding, the land is flat with elevations ranging from 105 to 65 m above sea level (a.s.l.). It has a continental monsoon climate, characterized by simultaneous rain and heat. According to the ground meteorological observation data from Shijiazhuang meteorological station in recent 60 years, the average annual precipitation is about 531 mm—mainly occurring during the summer season—and the average annual temperature is about 13.8°C.^1^ These natural conditions are beneficial to the development of agriculture. The main modern crops in Zhengding are grown in two different seasons, the first being winter wheat (*Triticum aestivum*), and the second being maize (*Zea mays*).^2^

Zhengding was an important Northern city in historical China, having been the seat of local government since the Han Dynasty (202 BCE–220 CE). The Yan mountains and their southern foothills were the natural barriers between the agrarian and nomadic regimes. During the Northern Song Dynasty (960–1127 CE), they were ruled by the Liao Dynasty (907–1125 CE), making Zhengding the front line for resistance to Liao cavalry and a place of strategic importance (the seat of *Hebeixilu*) for the Northern Song government. The Jin Dynasty, founded in northeastern China in 1115 CE, destroyed the Liao Dynasty in 1125 CE and occupied north-central China during 1125–1140 CE. Zhengding was captured by the Jin Dynasty in 1126 CE (Zhang, 1992) and retained its position as the seat of *Hebeixilu*.

**FIGURE 2:**
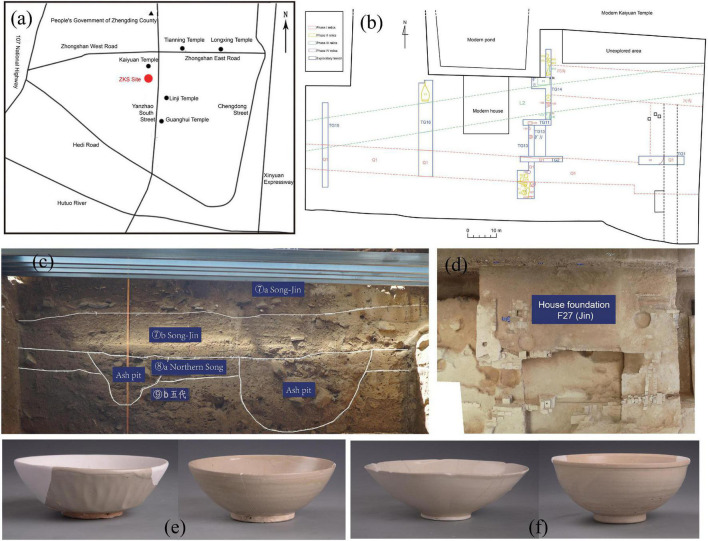
Images of the archeological sites and their cultural relics. **(a)** Location map of ZKS site; **(b)** general plan of the archaeological survey relics of the ZKS site; **(c)** relationship of cultural layers; **(d)** house foundation of F27; **(e)** porcelain bowls of the Northern Song Dynasty; **(f)** porcelain bowls of the Jin Dynasty.

Zhengding Kaiyuan Temple South has been excavated for 6 years since 2015 by the Hebei Provincial Institute of Cultural Relics and Archaeology. It is the first large-scale urban archaeological site in Hebei Province. The site covers a total area of nearly 12,000 and 3,664 m^2^ was systematically excavated. A large number of residential, artisanal, and commercial remains occurred in the Northern Song and Jin layers of ZKS site, which accounted for half of the total relic units, showing that both ordinary life and business characterized this period at ZKS site (Hebei Provincial Institute of Cultural Relics and Archaeology, 2019). Based on archaeological typology, layers 8, 7, and 6 at ZKS are dated to the Northern Song Dynasty, a period of coexistence between the Northern Song and Jin Dynasties, and the Jin Dynasty, respectively. The materials analyzed in this study were excavated from cultural layers, house foundations, and ash pits of layer 8 (Northern Song), layer 7 (Song-Jin), and layer 6 (Jin) (Figure 2).

## Materials and methods

### Flotation

In all, 66 samples (9 for layer 8, 34 for layer 7, and 23 for layer 6), totaling 382 liters, were collected for flotation analysis. Plant remains were obtained using 0.2-mm aperture sieves to collect the light fraction, and 1.25-mm aperture sieves to collect heavy objects. After drying and sorting through 0. 35-, 0. 7-, 1-, 2-, and 4-mm mesh sieves, all caryopses were selected using a 40 × stereo microscope. Carbonized plant caryopses were identified in the Paleoethnobotany Laboratory, Institute of Archaeology, Chinese Academy of Social Sciences.

### Obtaining an accurate sequence by accelerator mass spectrometry ^14^C dating and bayesian chronological modeling

Nine charred crop samples (wheat and foxtail millet grains) were selected for accelerator mass spectrometry (AMS) ^14^C dating. The crop AMS ^14^C samples were prepared using standard pre-treatment (acid-alkali-acid) and measured at the MOE Key Laboratory at Lanzhou University. All dates were calibrated by the OxCal v4.4.4 program (Ramsey, 2009), using the IntCal 20 Atmospheric curve in all calculations (Reimer et al., 2020). Based on AMS ^14^C dating, ceramic dates from the site, and written records, Bayesian modeling of the ^14^C chronological data was performed using the built-in “Phase” function of the OxCal online program1 and IntCal20 curves (Reimer et al., 2020), using the “R_Date” function to enter the ^14^C dates. Each “Phase” included all ^14^C dates of a culture layer, and each “Phase” function was bounded by the “Boundary” function. The start and end time of each culture was constrained using this function, and the “Order” function was used to order the beginning and end of each culture. Furthermore, the “outlier” function was used for each ^14^C chronological measurement (Long et al., 2017), to ensure that the model was reliable (Ramsey, 2009). The Bayesian model results were reported as a range of 95.4 and 68.3% and the median-to-median range was used to determine the chronological range of different cultures (Long and Taylor, 2015; Guo et al., 2018).

### Archaeobotanical analysis and yield estimation

Frequency (percentage or proportion of total crop remains) and ubiquity (number of samples in which it occurred) is the most commonly used measures for analyzing the structure of past agricultural activities in a targeted area (Pearsall, 2000; Zhao, 2010). Furthermore, there were significant differences between the weight of the grains of particular crop species, and the percentages of charred caryopses were not necessarily a close reflection of the actual yield of grains produced. The quantitative methods used to estimate the yield percentage (weight) of crops in the reference works of Shen (1955) and Zhou et al. (2016) have, therefore, been extensively employed (Sheng et al., 2018; Chen T. et al., 2020; Yang et al., 2021; Li et al., 2022; Shi et al., 2022). The function is as follows:


$$P\,\left(s\right)=\frac{N\,s\,\times \,F\,s}{N\,1\,\times \,F\,1+N\,2\,\times \,F\,2+N\,3\,\times \,F\,3+N\,4\,\times \,F\,4+N\,5\,\times \,F\,5}$$

Here, *N*1 = number of foxtail millet grains, *F*1 = 2.6, *N*2 = number of wheat grains, *F*2 = 35, *N*3 = number of broomcorn millet grains, *F*3 = 7.5, *N*4 = number of barley grains, *F*4 = 45, *N*5 is the number of rice grains, *N*6 = 26, and *P* (*s*) = actual yield percentage of that particular crop (Shen, 1955; Zhou et al., 2016; Sheng et al., 2018).

### Multidisciplinary analysis

Historical documents and studies, as well as paleoclimate records and published archaeobotanical (macrofossil) data from relevant sites in different regimes in China during the 10th–13th centuries, were also reviewed. The study sites were plotted on a map made in Arc GIS10.2 based on longitude and latitude reported in the papers or estimated coordinates from other locality details provided; open access files obtained from GIS datasets for the approximate distributions of Chinese dynasties at Harvard University were also shown in the map.^3^

## Results

### Radiocarbon dating

**FIGURE 3:**
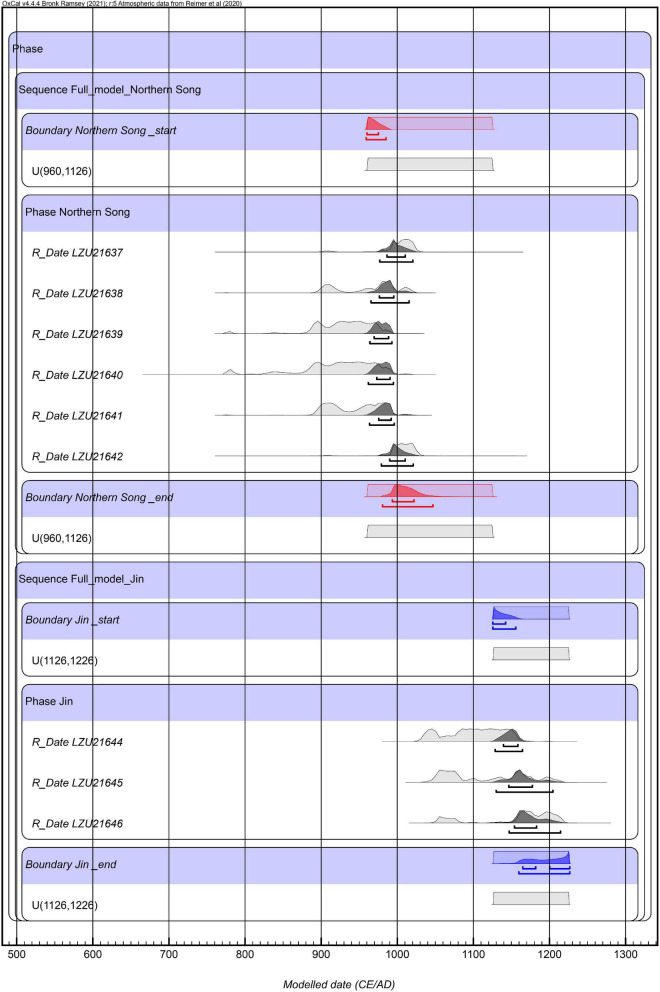
Radiocarbon dates from ZKS site, 2020 excavation calibrated in a Bayesian model incorporating stratigraphy and phasing, using OxCal 4.4 software.

Radiocarbon dates of crop remains are shown in Table 1. Two radiocarbon dates from culture layer 8 and the ash pit sealed by layer 8 were dated to around 895–1029 CE (LZU-21637, 21638). Two radiocarbon dates from culture layer 7 and two dates from the ash pit sealed by layer 7 were dated to around 774–1031 CE (LZU-21639, 21640, 21641, 21642). Two dates from culture layer 6 and one date from the house foundation sealed by layer 6 were dated to around 1033–1220 CE (LZU-21644, 21646, 21645). According to the historical documents, the reigning period of the Northern Song (960–1126 CE) and Jin (1126–1226 CE) Dynasties in Zhengding (Zhang, 1992) correspond to the radiocarbon dates of layers 8 and 6, respectively. All the ranges of the radiocarbon dates from layer 7 were before 1126 CE, indicating that plant remains from layer 7 came from the Northern Song Dynasty. Our analysis suggests that layer 8 and layer 7 were formed at the same time during the Northern Song Dynasty, while layer 7 in the upper stratum was disturbed and mixed with remains from the Jin Dynasty in the later period. The results from the Bayesian model (Figure 3) are given for the Northern Song Phase (968 CE∼1008 CE) and Jin Phase (1134 CE∼1195 CE).

**TABLE 1:** Radiocarbon dates from ZKS site. Calibrated ranges are given at 68.2 and 95.4% probabilities using OxCal 4.4 and the IntCal20 curve.

Context	Material	Lab Code	Cal. date BP	Calibrated age CE (68.2% prob.)	Calibrated age CE (95.4% prob.)
H80 (sealed by layer 8)	Foxtail millet grains (*n* = 15)	LZU21637	1050 ± 20	994–1021	904–1029
Layer 8	Wheat grain	LZU21638	1080 ± 20	899–1014	895–1021
Layer 7	Wheat grain	LZU21639	1130 ± 20	890–973	882–991
Layer 7	Wheat grain	LZU21640	1130 ± 30	889–976	774–994
HI1 (sealed by layer 7)	Wheat grain	LZU21641	1100 ± 20	898–991	892–994
HII (sealed by layer 7)	Wheat grain	LZU21642	1040 ± 20	999–1021	989–1031
Layer 6	Foxtail millet grains (*n* = 15)	LZU21644	950 ± 20	1040–1151	1033–1158
F27 (sealed by layer 6)	Foxtail millet lump	LZU21645	910 ± 20	1048–1199	1043–1212
Layer 7	Wheat grain	LZU21646	890 ± 20	1158–1212	1048–1220

### Crop assemblages in the Zhengding Kaiyuan Temple South site under the Northern Song and Jin Regimes

**FIGURE 4:**
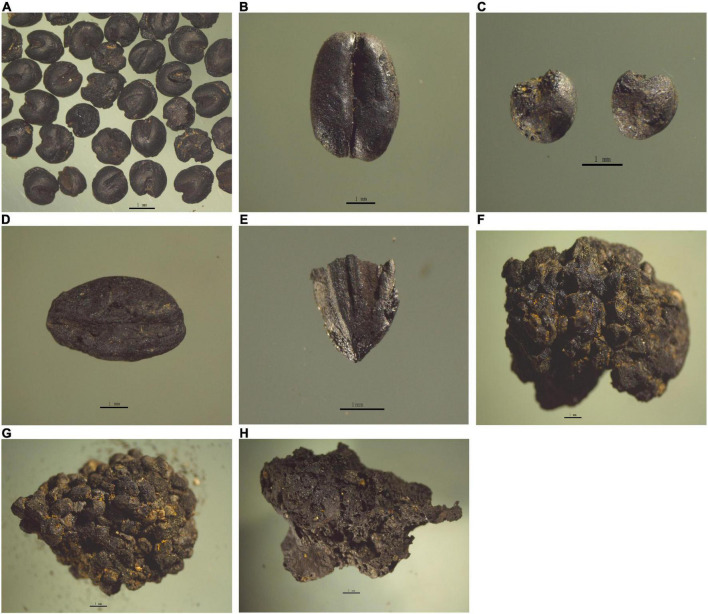
Domestic crops recovered from ZKS site. **(A)** Foxtail millet; **(B)** wheat; **(C)** broomcorn millet; **(D)** hulled barley; **(E)** rice; **(F–H)** boiled plant containing foxtail millet caryopses.

A total of 21,588 charred crop caryopses were identified from the 382 flotation samples collected. These included five types (Table 2 and Figure 4): 21,413 foxtail millet (*Setaria italica*) caryopses, 133 wheat (*Triticum aestivum*) caryopses, and 39 broomcorn millet (*Panicum miliaceum*) caryopses, two hulled barley (*Hordeum vulgare*) caryopses, and one rice (*Oryza sativa*) caryopsis. Charred foxtail millet was the most common grain, while wheat ranked second among all crops identified.

**TABLE 2:** Results of counts of crop remains in the floatation samples from ZKS site.

Samples	Northern Song	Song-Jin	Jin*
Volume flotated (liters)	Around 52L	Around 240L	Around 90L
No. of samples	9	34	23
No. of identified crops			
Foxtail millet	88	315	21010
Wheat	8	79	46
Broomcorn millet	3	14	22
Hulled barley	0	2	0
Rice	1	0	0
Total crop remains	100	410	21078

*A large number of crop remains have been unearthed from two flotating samples of the Jin Dynasty, including 353.5 g foxtail millet and 55.15 g boiled plant containing foxtail millet caryopses.

**FIGURE 5:**
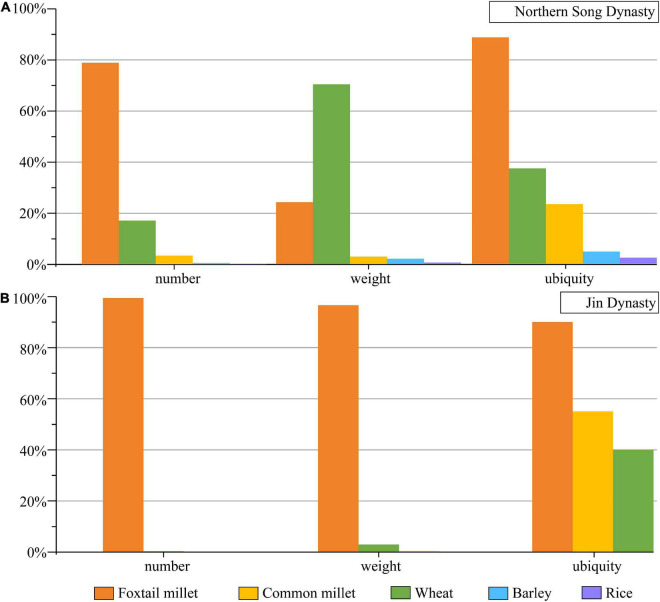
Comparison of number proportion, weight proportion, and ubiquity of different crops in **(A)** Northern Song Dynasty and **(B)** Jin Dynasty in ZKS site.

Based on the new radiocarbon dates and archaeobotanical data, we can draw a summary of crop utilization in the Zhengding area during the Northern Song and Jin Dynasties. The percentage, weight proportion, and ubiquity of different crops are not equivalent in different periods (Table 2 and Figure 5). Five types of crops were recorded from the Northern Song Dynasty: 403 foxtail millet caryopses (79.0% by number; 24.2% by weight), 87 wheat caryopses (17.1% by number; 70.2% by weight), 17 broomcorn millet caryopses (3.3% by number; 2.9% by weight), two hulled barley caryopses (0.4% by number; 2.1% by weight) and one rice grain (0.2% by number; 0.6% by weight), indicating mixed agriculture based on dry farming of foxtail millet and wheat. According to the estimated actual yield percentage, it is likely that wheat was the predominant crop, and foxtail millet ranked second, supplemented by broomcorn millet and hulled barley. By comparison, rice seemed to have a little effect based on this result. According to written records, a large number of artificial ponds were constructed for resisting the cavalry of the Liao Dynasty, and these provided conditions for the growth of rice, although its cultivation seemed to be poorly managed and had a limited role in human livelihoods (Han, 1993).

Three crop types were discovered in the ZKS site from the Jin Dynasty. Foxtail millet played the dominant role with 21,020 caryopses accounting for 99.7% by number and 96.8% by weight. As supplements to the foxtail millet, 46 wheat caryopses and 22 broomcorn millet caryopses accounted for 0.2 and 0.1% by number, and 2.9 and 0.3% by weight, respectively. In addition, 353.5 g of foxtail millet caryopses and 55.15 g of boiled porridge made of foxtail millet, although not involved in the proportion in order not to affect the statistical results, provided further evidence for foxtail millet being the dominant stable food in the livelihood of people.

## Discussion

### Agricultural patterns under different regimes in China during the 10th–13th centuries

**FIGURE 6:**
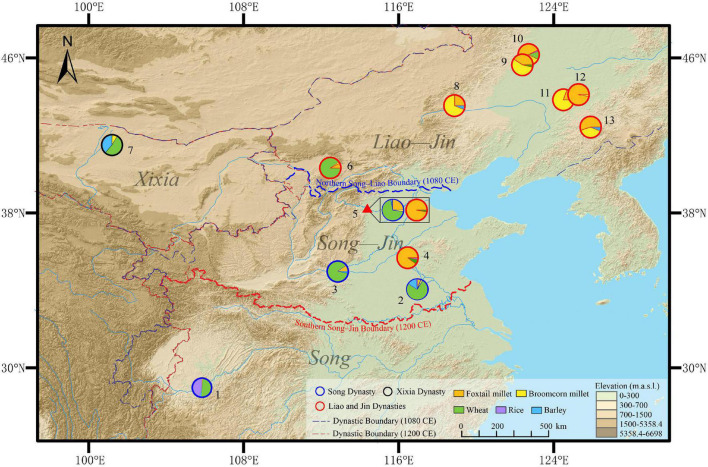
Comparison of estimated actual yield proportions of ZKS and other sites: 1. Handong Cheng (Ma et al., 2020); 2. Oupanyao (Bai et al., 2017); 3. Xijincheng (Chen et al., 2010); 4. Liang Zhuang (Wu et al., 2010); 5. ZKS (this study); 6. Zhuangwocun (Zhao, 2020); 7. Heicheng (Shi et al., 2022); 8. Bayantala (Sun and Zhao, 2014); 9. Sun Changqing (Yang et al., 2010a); 10. Yongping (Yang, 2014a); 11. Changshan (Chen, 2019); 12. Lichunjiang (Yang et al., 2010b); 13. Luotongshancheng (Yang, 2014b).

Multiple regimes established by agrarian and nomadic peoples existed in China from the 10th to the 13th centuries and engaged in significantly different livelihoods during the period. To achieve a better understanding of the cropping patterns under different ethnic regimes, we reviewed macrobotanical data and written records from different regimes during the 10th–13th centuries in China, including the Song (960–1127 CE of Northern Song and 1127–1279 CE of Southern Song), the Jin (1115–1234 CE), the Liao (907–1125 CE), and the Xixia (1038–1227 CE) Dynasties (Figure 6).

The archaeobotanical analysis of ZKS shows that wheat was the dominant staple food in the Northern Song, and we found that it was similar to other sites under the control of the Song and the Xixia regimes (Figure 6). The sites of Xijincheng in Henan Province and Oupanyao in Anhui Province, dated to the Tang and Song Dynasties, produced 34 and 318 wheat caryopses, respectively, accounting for 75 and 87% of the total yield of crops, with millets and barley as supplements (Chen et al., 2010; Bai et al., 2017). Located on the upper reaches of the Yangtze River, the Handong Cheng site remained under the control of the Song Dynasty (960–1279 CE) during the 10th–13th centuries, producing 155 millet caryopses, 380 wheat caryopses, 478 rice caryopses, and other plant caryopses (Ma et al., 2020), indicating that wheat (50% of the yield percentages) and rice (46% of the yield percentages) were the dominant crops. According to the written records, a two-tax law was adopted in the Song Dynasty (960–1279 CE), with wheat or cash crops paid in the summer and foxtail millet or rice paid in the fall (Ma, 2012), showing that the double staple grain model was implemented, with wheat being the main grain in both the north and the south (Min et al., 1983; Han, 2013). Neighboring the Northern Song Dynasty, the Xixia Dynasty (1038–1227 CE) also maintained wheat-based agriculture with wheat and barley caryopses contributing 52 and 39% in the yield percentages recovered from Heicheng site in the lower reaches of Hei River (Shi et al., 2022). In addition, wheat was also recorded as the main taxable crop in Hexi corridor during the Xixia Dynasty (Wang, 1982; Li, 2002; Ma, 2012).

The archaeobotanical results from ZKS indicate that the dominant staple food changed to foxtail millet in the Jin period, with most sites under the Jin regime in northeastern and north-central China showing the same trend, except one in the agro-pastoral region of the Loess plateau. Six original sites from the Liao and Jin Dynasties show that millets were the dominant crops during the 10th–13th centuries. They had 300, 1133, 931, 830, 3146, and 418 carbonized millets caryopses identified (Yang et al., 2010a,b; Sun and Zhao, 2014; Yang, 2014a,b; Chen, 2019), contributing 100% of the yield percentage in two of the sites, and more than 85% in four of the sites which were supplemented by a few wheat and barley caryopses. According to the written records and zooarchaeological data, the *Jurchen* lived a life of millet-based agriculture, together with pastoralism, fishing, and hunting in the native land of the Jin Dynasty (Han, 1999; Liang et al., 2018). After the regime in north-central China changed to the Jin Dynasty, 21,032 and 49,980 carbonized millet caryopses accounting for over 90% of the yield percentages occurred in the ZKS (this study) and Liang Zhuang (Wu et al., 2010) sites, which were located in Hebei and Shandong Provinces, respectively. In addition, the boiled foxtail millet unearthed from ZKS site was identified as carbonized porridge, which also occurred in other city sites during the Liao and Jin Dynasties (Institute of Archaeology, Chinese Academy of Social Sciences, 2022). This suggested that porridge made of foxtail millet was commonly eaten by people living in the Jin Dynasty. However, Zhuangwocun, located in the agro-pastoral region of the Loess plateau, produced 422 wheat caryopses and spike-stalks in the Liao and Jin periods (Zhao, 2020), contributing 90% of the yield percentage. Combined with 301 forage weed caryopses, this suggests that wheat-based agriculture and pastoralism were the main livelihoods in that region (Zhao, 2020). In addition, scenes of agricultural and pastoral lifestyles coexisted in frescoes of the Liao and Jin Dynasties in Datong, indicating an agro-pastoral economy there (Wang, 2021).

In summary, wheat-based agriculture was carried out in north-central China during the Northern Song (960–1127 CE) Dynasty. Wheat-barley and wheat-rice cropping systems were used in northwestern and southwestern China under the Xixia (1038–1227 CE) and Song (960–1279) regimes, respectively. In the Jin Dynasty (1115–1234 CE), the agricultural condition was complex. The native residents of the Jin Dynasty were engaged in millet-based agriculture in northeastern China. North-central China experienced regime change from the Northern Song to Jin in the 12th century, during which the dominant staple food transformed from wheat to foxtail millet. At the same time, people in the agro-pastoral region of the Loess plateau adopted a lifestyle of wheat-based agriculture and pastoralism during the Liao and Jin periods.

### Factors influencing agricultural systems in China during the 10th–13th centuries

**FIGURE 7:**
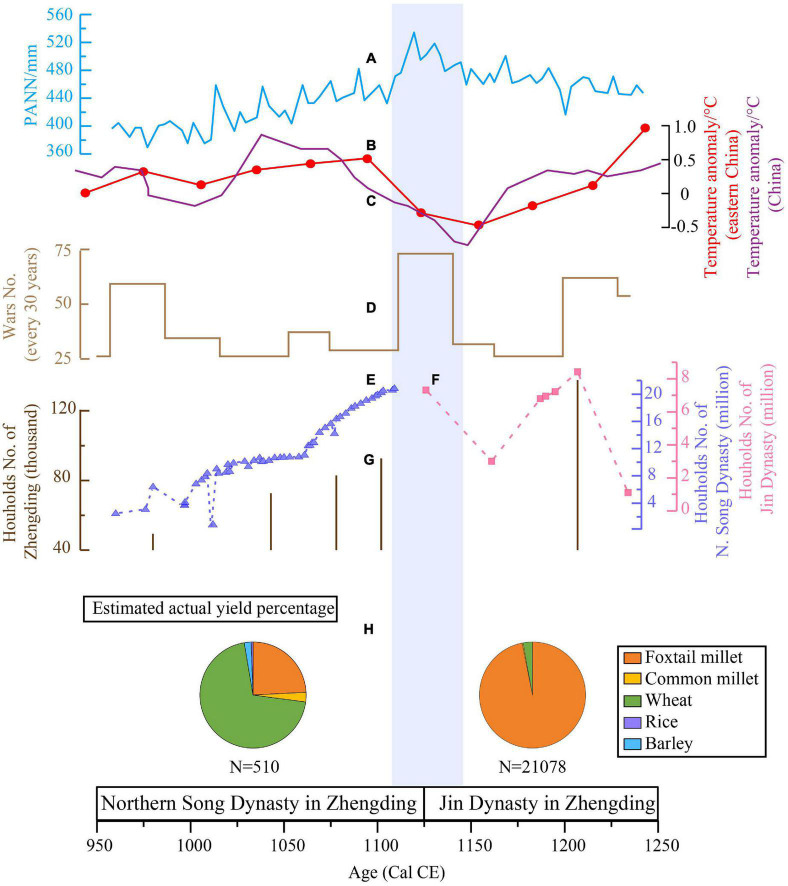
Comparison of estimated actual yield percentage **(H)** of major crops with historical and climatic records: **(A)** Reconstructed PANN from Gonghai lake (Chen et al., 2015b); **(B)** winter half-year temperature anomaly change in eastern China (Ge et al., 2003); **(C)** China-wide temperature composites established by combining multiple paleoclimate proxy records (Yang et al., 2002); **(D)** war number (Ge et al., 2014); Households number of **(E)** Northern Song and **(F)** Jin dynasties and **(G)** Zhengding prefecture (Wu, 2000). The blue strip represents the cold event in the 12th century.

Scholars have demonstrated that the transformation of human livelihoods can result from past changes in both climate and society (Chen et al., 2015a; Ma et al., 2016; Zhou et al., 2016; Dong G. H. et al., 2017; Li R. et al., 2020). To explore the variables behind changing agricultural systems of humans in the historic period, we produced a PANN (annual mean precipitation) reconstruction from lacustrine deposits of the Gonghai Lake in Northern China (Chen et al., 2015b; Figure 7A); a half-year winter temperature reconstruction from phenological cold/warm events recorded in Chinese historical documents in eastern China (Ge et al., 2003; Figure 7B), China-wide temperature composites established by combining multiple paleoclimate proxy records obtained from ice cores, tree rings, lake sediments and historical documents (Yang et al., 2002; Figure 7C); a count of the number of wars (The Compilation Group of Military History of China, 2003; Ge et al., 2014; Figure 7D), and a population scale for the Northern Song and Jin Dynasties (Wu, 2000; Figures 7E–G). These were compared with the archaeobotanical results (Figure 7H).

### The impact of geopolitics on the transformation of agricultural systems

Changes in the geopolitical patterns in historical periods have been demonstrated to significantly affect the agricultural structure (Messer, 1997; Han, 1999; Wilson et al., 2022). There were multiple powerful regimes existing in Northern China during the 10th–13th centuries, with frequent conflicts between regimes and significant changes in their territories, which might be related to the changing situation of agricultural systems in this period (Han et al., 2012; Ma et al., 2020; Shi et al., 2022). The Northern Song was founded in 960 CE, neighboring the Liao Dynasty (907–1125 CE) to the north and the Xixia Dynasty (1038–1227 CE) to the west, with frequent wars in the early years (Ge et al., 2014; Figure 7C). Since the signing of the Chanyuan Alliance (*澶渊之盟*, the peace treaty between the Northern Song and Liao dynasties) in 1005 CE, a society under the Northern Song Dynasty was stabilized, promoting rapid population growth and increasing demand for food (Wu, 2000; Figure 7D), which might have enhanced the importance of high-yield wheat. According to written records, water conservancy facilities for artificial irrigation increased frequently, and the times of repair reached 10,793 in 7 years of the reign of emperor *Song Shenzong* (Zhang, 1990). Techniques of wheat cultivation and wheat-based cooking also developed markedly in the Northern Song Dynasty, with steamed stuffed buns, wheat cakes, and noodles occurring frequently in the texts (Meng and Deng, 1982; Wang, 2016). The population pressure, together with the development of planting and cooking techniques may, therefore, have helped promote the establishment of wheat-based agriculture in the Northern Song Dynasty.

During 1115–1141 CE, frequent wars took place between Liao, Jin, and Northern Song Dynasties in northeastern and north-central China, which was later occupied by the Jin Dynasty. These conflicts resulted in economic losses and mass migration from north-central China. On the one hand, migrants might have brought advanced techniques for cultivating and cooking wheat to areas such as the south and northwest (Wu, 2000; Li, 2002), ultimately leading to the formation of wheat agriculture in these areas where the previous dominant crops were rice and foxtail millet, respectively (Han et al., 2012; Ma et al., 2020; Shi et al., 2022). On the other hand, to restore the business and consolidate their rule, the Jin government set north-central China as a key area for management, and encouraged the immigration of about three million *Jurchen* people from the northeast, providing them with fertile land and agricultural tools (Xie, 1986; Liu, 1994; Han, 1999). Based on demographic calculations, 73.1% of the immigrant *Jurchen* people were placed in Hebei and Shandong provinces (Liu, 1994). The land given to the *Jurchen* people amounted to about one-third of the agricultural land in the region (Han, 2000), which might have been a shock to the resident people and their agriculture. Moreover, the Jin government targeted foxtail millet, the major crop grown in the native land of the *Jurchen*, as the most common taxation source, and relied on it for army provisions, official salary, and granaries (Han, 1999). Inevitably, the *Han* people were influenced and converted to foxtail millet as the main crop for their livelihoods. For example, a bowl of foxtail millet appeared in a *Han* tomb from the Jin Dynasty (Chang, 1959), and carbonized porridge made of foxtail millet was a common food on sites of the Jin Dynasty (this study; Institute of Archaeology, Chinese Academy of Social Sciences, 2022), In addition, the archaeobotanical data in this study and Wu et al. (2010) found a simultaneous shift of the dominant crop from wheat to foxtail millet in north-central China.

Because foxtail millet has a lower yield and nutritional value than wheat (Monfreda et al., 2008; Mueller et al., 2012; D’Alpoim and Butler, 2014), it seemed unusual for the dominant crop to shift from wheat to foxtail millet. In the Loess plateau, which was not a traditional agricultural area, there was no resettlement of migrants by the Jin government, and the area continued to maintain livelihoods based on wheat and pastoralism (Zhao, 2020; Wang, 2021). The agricultural districts like *Hebeilu* in north-central China, which was one of the top tax-paying areas, produced large supplies of foxtail millet, which were transported to densely populated cities like *Yanjing* (Beijing) and *Bianjing* (Kaifeng) for consumption (Xie, 1990; Han, 1999). We suggest, therefore, that government policies on immigration and taxation, which were in favor of the *Jurchen* people and their native crop, resulted in the changes to the agricultural systems and human livelihoods in north-central China during the Jin Dynasty. The change of regimes in Northern China also led to agricultural transitions toward wheat in the south and northwest due to mass migration bringing technologies of wheat cultivation and processing. What is puzzling is why did the *Jurchen* choose to maintain the distinctive agriculture based on millets in the 10th–13th centuries?

### Relationship between the natural environment and agricultural systems in Northern China

Previous studies have shown that geopolitics is not the only factor influencing agricultural patterns in the past, environmental factors also played an important role (Chen et al., 2015a; Ma et al., 2016; Zhou et al., 2016; Dong Y. et al., 2017). There are various natural environmental conditions in Northern China, such as the higher latitudes having lower temperatures, which have a critical impact on the geographical distribution and yield of crops. With simultaneous rain and heat, and a relatively long frost-free period, north-central China provides favorable conditions for agricultural development and therefore has a long agricultural history (Yang et al., 2012). However, northeastern China is located between latitudes 40°N–53°N, with extremely long cold winters, and short summers suitable for plant growth. The modern crop growing season in the northeast is about 130–210 days, compared to the growing seasons of 240–270 days in north-central China, so the duration of crop growth was crucial for agriculture in the northeast. The maturation period of wheat is 230–270 days, while millets which only need 80–120 days to mature (Weber et al., 2010) are more suitable for growing in the northeast than wheat. There were other short-term crops, like soybean, sorghum, and cultivated barnyard millet, which meet the mobile livelihood style of nomadic *Jurchen* people, widely regarded as the native crops of the *Jurchen* according to the written records and archaeobotanical data (Han, 1999; Yang et al., 2010a, Yang, 2014a). Moreover, wheat needs 800–1,100 mm average annual rainfall, which is two or three times more than millets (Shrestha et al., 2017). Reconstructions from Gonghai Lake pollen cores indicate that the average annual precipitation in Northern China ranged between 370 and 535 mm in the 10th–13th centuries (Chen et al., 2015b; Figure 6), meaning that artificial irrigation would be needed for wheat cultivation there. The *Jurchen* maintained a slave-like society before the invasion of central China, so limiting the potential for social organization, which might impede the construction of the labor-intensive irrigation facilities, as well as the development of wheat cultivation (Xie, 1990; Han, 1999). A humid and semi-humid climate due to low evaporation made northeastern China rich in natural resources, providing the *Jurchen* with the basis for mixed livelihoods based on fishing, hunting, pastoralism, and agriculture (Han, 1999, 2000). However, a significant cooling in the 11th–12th century in Northern China can be observed in the phenological records and the reconstructed temperature established by paleoclimate proxy records (Zhu, 1973; Yang et al., 2002; Ge et al., 2003; Marcott et al., 2013). For example, the two snow disasters in 1078 CE and 1126 CE were listed as the most serious strong cold wave disasters in the Northern Song Dynasty (Wen and Ding, 2008). The cold events might have affected the production of natural resources and frost-sensitive millet agriculture in the northeast, as well as the boundaries of the agro-pastoral intersection (Chen F. H. et al., 2020), and perhaps have been one of the reasons for the wars launched by the Jin Dynasty over agricultural resources. Eventually, the traditional dietary preference for foxtail millet in *Jurchen* society promoted the transformation of human livelihoods from wheat-based to foxtail millet-based agriculture in north-central China.

Combined with previous studies on the development of agriculture in ancient China, our findings show that the conversion from foxtail millet to wheat was a complex process influenced by several factors, including natural and geopolitical variables (Han, 1999; Ma et al., 2016; Li X. et al., 2020, Li et al., 2022; Shi et al., 2022). Because of the different growth characteristics of wheat and foxtail millet (wheat is frost-tolerant but drought-sensitive while foxtail millet is drought-tolerant but frost-sensitive), wheat was first grown as a supplement to foxtail millet, driven by food scarcity triggered by climate change, especially the cooling event (Ma et al., 2016; Zhou et al., 2016, 2017; Dong Y. et al., 2017; Li X. et al., 2020). The shift to greater acceptance of wheat occurred in the Wei and Jin dynasties (220–420 CE) as well as the Tang and Song dynasties (618–1279 CE). These are periods in Chinese history when massive population migration took place and contributed to the spread of wheat processing and farming technologies, thus expanding the land under wheat cultivation (Lin, 1984; Han, 1999; Bao and Li, 2015; Shi et al., 2022). Moreover, the implementation of two taxes levied in summer and autumn since the Tang Dynasty might have reflected rotations of winter wheat with foxtail millet or rice, offering important evidence for the establishment of wheat as the dominant crop (Yan and Chen, 2012; Han, 2013; Wang, 2016). To this extent, the progression of wheat was controlled by socio-political and technological factors. However, in the target region of north-central China, we find that although wheat took over dominance from foxtail millet during the Northern Song Dynasty (960–1127 CE), the dominant crop could turn back to foxtail millet under the nomadic regime of Jin (1115–1234 CE), reminding us that there were significant dietary differences between farming and nomadic peoples. The development of agriculture in historic China is a complex process affected by environmental conditions, dietary tradition, climate change, and most directly in this case study, geopolitics.

## Conclusion

In this paper, we present nine AMS ^14^C dates obtained from short-lived plant remains, combined with ceramic and historical dates, as well as establish an accurate chronology for the Northern Song and Jin layers of the ZKS site. The results show that the plant remains of layers 8 and 7 belonged to the Northern Song Dynasty (960–1127 CE), and plant remains of layer 6 were from the Jin Dynasty (1115–1234 CE). The archaeobotanical analysis also shows that the dominant crop for people’s livelihoods altered from wheat to foxtail millet during the regime change from the Northern Song to the Jin dynasties in Zhengding. In comparison to historical documents and paleoclimate records, this abnormal shift was induced by the Jin government policies on immigration and land taxation, which favored the *Jurchen* people and their native crops. In addition, a significant cooling in the 12th century in Northern China might have disrupted the frost-sensitive millet agriculture in the northeast and resulted in wars against north-central China for agricultural resources. In summary, we propose that the primary influencing factor for the transformation of human livelihoods in north-central China during the 10th–13th centuries was geopolitics and that climate change indirectly affected this process.

## Data availability statement

The original contributions presented in this study are included in the article/supplementary material, further inquiries can be directed to the corresponding authors.

## Author contributions

YL and BL designed the study. RL, WC, PL, and MX conducted field surveys and sample collection. RL, PL, MX, and SW completed experiments and data correction. RL, BL, WC, and YZ analyzed data and designed the figures. RL, BL, and PL wrote the manuscript in consultation with all authors. All authors discussed the results and commented on the manuscript.

## Footnotes

1
http://data.cma.cn/

2
http://www.zd.gov.cn/col/1584426346749/index.html

3
https://sites.fas.harvard.edu/~chgis/data/hartwell/
